# Neurovascular Coupling in Hypertension Is Impaired by IL-17A through Oxidative Stress

**DOI:** 10.3390/ijms24043959

**Published:** 2023-02-16

**Authors:** Jessica Youwakim, Diane Vallerand, Helene Girouard

**Affiliations:** 1 Département de Pharmacologie et Physiologie, Université de Montréal, Montreal, QC H3T 1J4, Canada; 2 Groupe de Recherche Universitaire sur le Médicament (GRUM), Montreal, QC H3C 3J7, Canada; 3 Centre Interdisciplinaire de Recherche sur le Cerveau et l’Apprentissage (CIRCA), Montreal, QC H3T 1P1, Canada; 4 Groupe de Recherche sur la Signalisation Neuronal et la Circuiterie (SNC), Montreal, QC H3T 1J4, Canada

**Keywords:** neurovascular coupling, angiotensin II, hypertension, interleukin-17A, NADPH oxidase 2, reactive oxygen species, oxidative stress, inflammation

## Abstract

Hypertension, a multifactorial chronic inflammatory condition, is an important risk factor for neurovascular and neurodegenerative diseases, including stroke and Alzheimer’s disease. These diseases have been associated with higher concentrations of circulating interleukin (IL)-17A. However, the possible role that IL-17A plays in linking hypertension with neurodegenerative diseases remains to be established. Cerebral blood flow regulation may be the crossroads of these conditions because regulating mechanisms may be altered in hypertension, including neurovascular coupling (NVC), known to participate in the pathogenesis of stroke and Alzheimer’s disease. In the present study, the role of IL-17A on NVC impairment induced by angiotensin (Ang) II in the context of hypertension was examined. Neutralization of IL-17A or specific inhibition of its receptor prevents the NVC impairment (*p* < 0.05) and cerebral superoxide anion production (*p* < 0.05) induced by Ang II. Chronic administration of IL-17A impairs NVC (*p* < 0.05) and increases superoxide anion production. Both effects were prevented with Tempol and NADPH oxidase 2 gene deletion. These findings suggest that IL-17A, through superoxide anion production, is an important mediator of cerebrovascular dysregulation induced by Ang II. This pathway is thus a putative therapeutic target to restore cerebrovascular regulation in hypertension.

## 1. Introduction

Hypertension is the most prevalent modifiable risk factor for neurovascular and neurodegenerative diseases, including stroke and Alzheimer’s disease [1]. Although hypertension treatments greatly reduce stroke incidence [2], their impact on cognitive dysfunction is less clear [3]. This underlies the importance of better understanding the mechanisms by which hypertension affects the brain.

In hypertensive humans and experimental models of hypertension, important alterations of cerebral blood flow (CBF) regulation, including neurovascular coupling (NVC), have been observed [4,5,6,7,8]. NVC is the dynamic link between neuronal activity and local blood supply. Its importance is of significance since the brain has high energy needs and no energy reserve; thus, slight alterations of this mechanism can negatively impact cerebral protein synthesis and neuronal functions [9]. Understanding the mechanisms underlying NVC impairment in hypertension is crucial to develop preventive approaches to preserve the brain’s health. Angiotensin (Ang) II, a peptide known to be involved in the development of hypertension, impairs NVC independently of blood pressure [5,6,10]. Interestingly, hypertension is now recognized as being a subclinical inflammatory condition, and Ang II is a powerful modulator of the immune system. In an experimental model of hypertension induced by chronic Ang II perfusion in mice, we previously revealed the impact of inflammation on NVC through anti-inflammatory treatments with T regulatory (Treg) lymphocytes (CD4+/CD25+) or interleukin (IL)-10 [4]. These treatments prevent gliosis and reactive oxygen species (ROS) production suggesting that the inflammatory conditions play a role in maintaining high ROS levels.

IL-17A was shown to be upregulated in many experimental models of hypertension and in hypertensive humans. In 2010, Madhur et al. reported that the pro-inflammatory cytokine IL-17A, through its IL-17A receptor, is required for the maintenance of hypertension induced by Ang II and that serum IL-17 levels are correlated with blood pressure in humans [11]. The IL-17 cytokine family is composed of six isoforms (going from IL-17A to IL-17F), where the biological function and regulation of IL-17A and IL-17F are best understood. Even though these two cytokines share the strongest sequence homology and both modulate pro-inflammatory responses, IL-17A, but not IL-17F, has been demonstrated to play a role in hypertension-associated end-organ damages for cardiac, vascular, and renal injuries [12,13,14]. In the brain, in a murine model of a high-salt diet, anti-IL-17A treatments prevent reduced resting CBF and impaired NVC [15]. However, whether IL-17A could impair NVC by itself or be involved in the NVC impairment induced by Ang II remains to be investigated.

Ang II impairs NVC by activating its AT1 receptor and nicotinamide adenine dinucleotide phosphate (NADPH) oxidase, specifically the NADPH oxidase (NOX) 2 subtype [5]. Interestingly, IL-17 induces vascular inflammation through NOX2-derived ROS production [16]. Thus, ROS production from NOX2 is a possible converging mechanism by which Ang II and IL-17A impair NVC. The hypothesis is that NVC impairment induced by chronic Ang II administration is mediated by IL-17A and the subsequent increase in NOX2-dependent superoxide production. To explore this question, we determined the impact of IL-17A on NVC, as CBF changes in response to whiskers stimulation with laser Doppler flowmetry and ROS production using an IL-17A neutralizing antibody (Ab) and an antagonist against its receptor. After establishing that recombinant (Rb) IL-17A can by itself impair NVC, chronic treatments with the ROS scavenger, Tempol, and NOX2 deletion were tested in mice chronically receiving IL-17A.

## 2. Results

### 2.1. Neutralization of IL-17A or Inhibition of Its Receptor Prevents the Ang II-Induced NVC Impairment

**Figure 1:**
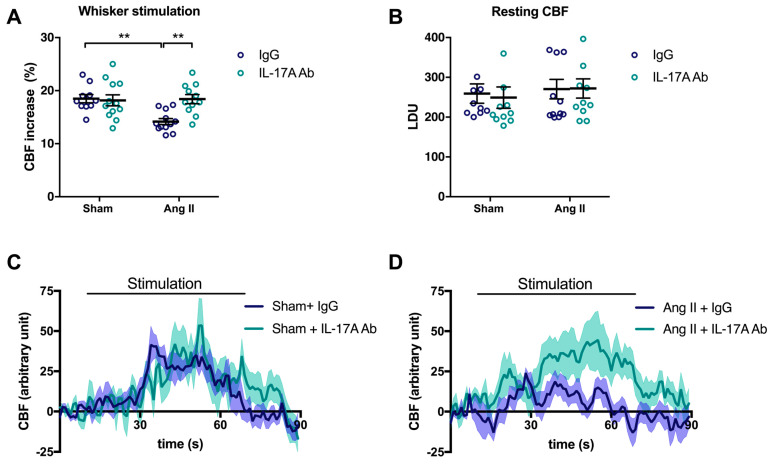
**IL-17A neutralization prevents neurovascular coupling impairment induced by Ang II.** Cerebral blood flow (CBF) in response to whiskers stimulations measured in vivo by laser Doppler flowmetry in C57BL/6 male mice treated with an anti-IL-17A neutralizing antibody (IL-17A Ab) or control mouse immunoglobulin G (IgG) antibody (0.5 μg/μL per mouse every 4 days, i.p.). This administration started at day 0 of Ang II (600 ng/kg/min, 14 days) infusion through an osmotic minipump or sham surgery. The graphs depict (**A**) CBF as percentage changes with respect to the initial CBF value, (**B**) resting CBF value as laser Doppler perfusion units (LDU), and (**C**,**D**) 1 s average curves of the evoked CBF, expressed in arbitrary unit. SEM is represented by the lighter tone shade surrounding each curve. Data were analyzed using an ANOVA for factorial design followed by a Bonferroni post-test. ** *p* < 0.01 and *n* = 10–12 per group.

To examine the involvement of IL-17A on NVC impairment induced by Ang II, injections of a neutralizing IL-17A Ab were administered in mice intraperitoneally (i.p.) every four days concomitantly with Ang II. As previously observed [6], Ang II attenuated CBF increases to 14.2 ± 0.6% in response to whiskers stimulations compared with 18.5 ± 0.8% in sham-operated mice (Figure 1A; *p* < 0.01). Chronic administration of the neutralizing IL-17A Ab prevented the NVC impairment induced by Ang II (Figure 1A,D; *p* < 0.01) without altering relative resting CBF (Figure 1B). However, it slightly attenuated the increase in systolic blood pressure (SBP) induced by Ang II at days 7 and 14 by 9.7 ± 3.0 and 9.4 ± 2.1 mmHg, respectively (Supplemental Figure S1). IL-17A Ab, on its own, did not elicit changes in cerebrovascular responses to neuronal stimulations (Figure 1A,C) or SBP (Supplemental Figure S1) in control mice.

**Figure 2:**
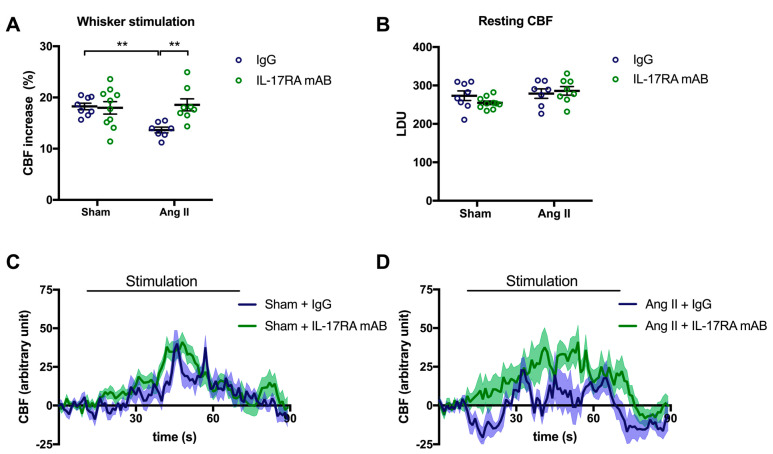
**IL-17A receptor inhibition prevents Ang II-induced neurovascular coupling alteration**. CBF in response to whiskers stimulations measured in vivo by laser Doppler flowmetry in C57BL/6 male mice treated with an IL-17A receptor antagonist (IL-17RA mAB) or control mouse IgG antibody (0.5 μg/μL per mouse every 4 days, i.p.). This administration started at day 0 of Ang II (600 ng/kg/min, 14 days) infusion through an osmotic minipump or sham surgery. The graphs depict (**A**) CBF as percentage changes with respect to the initial CBF value, (**B**) resting CBF value as LDU, and (**C**,**D**) 1 s average curves of the evoked CBF, expressed in arbitrary unit. SEM is represented by the lighter tone shade surrounding each curve. Data were analyzed using an ANOVA for factorial design followed by a Bonferroni post-test. ** *p* < 0.01 and *n* = 7–10 per group.

To assess whether IL-17A impairs NVC through its receptor, mice received an IL-17A receptor antagonist (IL-17RA mAB) simultaneously with the chronic systemic administration of Ang II. In this experimental group, CBF increased in response to whiskers stimulations by 13.6% ± 0.6 and 18.3 ± 0.6% in the presence or the absence of Ang II, respectively (Figure 2A; *p* < 0.01). Inhibiting IL-17RA prevented the NVC attenuation induced by Ang II (Figure 2A,D; *p* < 0.01) without modifying the relative resting CBF (Figure 2B). Nevertheless, inhibition of the IL-17A receptor attenuated the increased SBP induced by Ang II by 8.2 ± 4.7 and 7.9 ± 2.6 mmHg on days 7 and 14, respectively (Supplemental Figure S2). In control mice, the IL-17RA mAB did not elicit changes in cerebrovascular responses (Figure 2A–C) or SBP (Supplemental Figure S2). 

### 2.2. Neutralization of IL-17A or Inhibition of Its Receptor Prevents the Superoxide Anion Production Induced by Ang II

**Figure 3:**
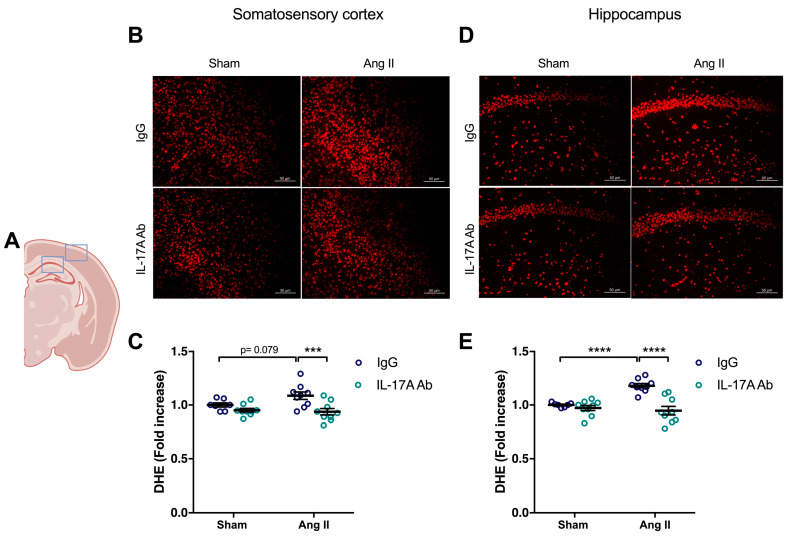
**IL-17A neutralization prevents Ang II-induced oxidative stress production**. Superoxide anion production quantified using dihydroethidium fluoromicrography (DHE) in C57BL/6 male mice treated with an IL-17A Ab or control mouse IgG antibody (0.5 μg/μL per mouse every 4 days, i.p.). This administration started at day 0 of Ang II (600 ng/kg/min, 14 days) infusion through an osmotic minipump or sham surgery. (**A**) Scheme illustrating the acquisition area representing the somatosensory cortex (right box) and the CA1 of the hippocampus (left box). Representative micrographs of DHE staining in (**B**) the somatosensory cortex and (**D**) the CA1 of the hippocampus (50 μm scale). The graphs depict the ratio of fluorescence compared to control mice in the (**C**) somatosensory cortex and (**E**) the hippocampus. Data were analyzed using an ANOVA for factorial design followed by a Bonferroni post-test. *** *p* < 0.001 and **** *p* < 0.0001; *n* = 7–9 per group.

To determine whether the increase in ROS production induced by Ang II is mediated by IL-17A, we investigated whether IL-17A Ab reduces superoxide anion production induced by Ang II. As shown in Figure 3, the increased production of superoxide anion by Ang II seen in the somatosensory cortex (*p* = 0.079) and in the hippocampus (*p* < 0.0001) was prevented following IL-17A Ab administration. 

**Figure 4:**
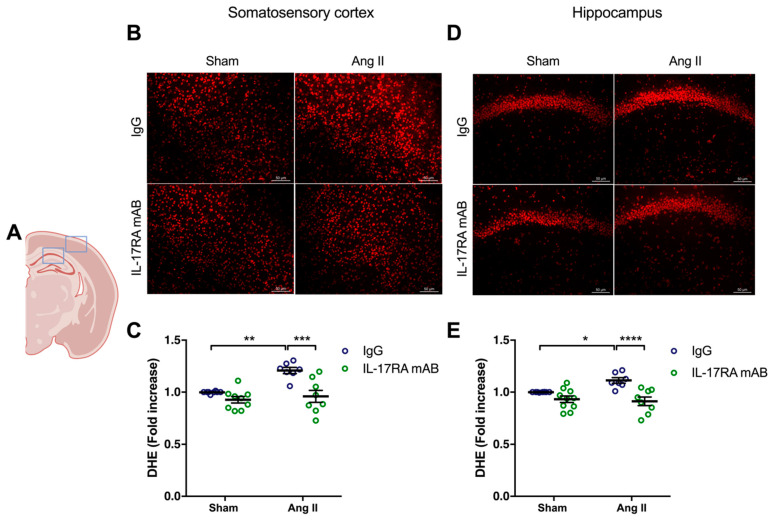
**IL-17A receptor inhibition prevents oxidative stress production induced by Ang II**. Superoxide anion production was quantified using DHE in C57BL/6 male mice treated with an IL-17RA mAB or control mouse IgG antibody (0.5 μg/μL per mouse every 4 days, i.p.). This administration started at day 0 of Ang II (600 ng/kg/min, 14 days) infusion through an osmotic minipump or sham surgery. (**A**) Scheme illustrating the acquisition area representing the somatosensory cortex (right box) and the CA1 of the hippocampus (left box). Representative micrographs of DHE staining in (**B**) the somatosensory cortex and (**D**) the CA1 of the hippocampus (50 μm scale). The graphs depict the ratio of fluorescence compared to control mice in (**C**) the somatosensory cortex and (**E**) the hippocampus. Data were analyzed using an ANOVA for factorial design followed by a Bonferroni post-test. * *p* < 0.05, ** *p* < 0.01, *** *p* < 0.001, and **** *p* < 0.0001; *n* = 7–9 per group.

Similarly, the higher production of superoxide anion in the somatosensory cortex (*p* < 0.01) and the hippocampus (*p* < 0.05) in mice receiving chronic administration of Ang II was prevented by IL-17RA mAB administration (Figure 4). In the control groups, the production of superoxide anion did not change after IL-17A Ab or IL-17RA mAB administration. 

### 2.3. Chronic Administration of an IL-17A Recombinant Impairs NVC

To demonstrate that IL-17A can, on its own, impair the cerebrovascular response, we evaluated whether the IL-17A Rb impairs NVC. Systemic IL-17A Rb administration reduces CBF increase in a dose-dependent manner (Supplemental Figure S3). At the selected dose of 50 pg/kg/h, IL-17A Rb administration reduced CBF increase in response to whiskers stimulations from 20.0 ± 1.1% in the sham group to 14.1 ± 1.1% (Supplemental Figure S3A; *p* < 0.01). As shown in Supplemental Table S1, IL-17A Rb administration led to a comparable plasmatic concentration to the one observed in Ang II hypertensive mice (7.66 ± 0.80 pg/mL, 11.12 ± 2.60 pg/mL, 13.43 ± 3.75 pg/mL in sham, IL-17A Rb, and Ang II, respectively). Interestingly, no change was observed in brain homogenates. 

### 2.4. Tempol Treatment or NOX2 Deletion Prevents Superoxide Anion Production and NVC Dysfunction Induced by IL-17A

**Figure 5:**
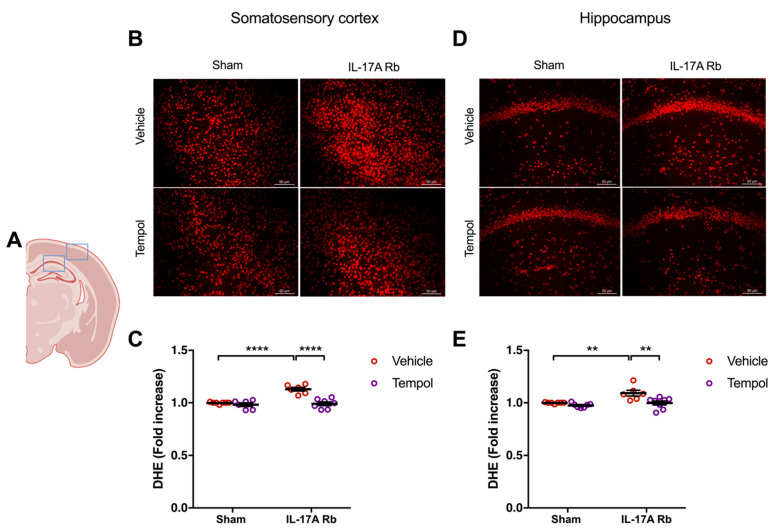
**Tempol treatment prevents IL-17A-induced oxidative stress production**. Superoxide anion production was quantified using DHE in C57BL/6 mice receiving IL-17A Rb through an osmotic minipump (50 pg/kg/h, 7 days) and treated with or without Tempol (1 mM changed every 2 days) administered in the drinking water. (**A**) Scheme illustrating the acquisition area representing the somatosensory cortex (right box) and the CA1 of the hippocampus (left box). Representative micrographs of DHE staining in (**B**) the somatosensory cortex and (**D**) the CA1 of the hippocampus (50 μm scale). The graphs depict the ratio of fluorescence compared to control mice in (**C**) the somatosensory cortex and (**E**) the hippocampus. Data were analyzed using an ANOVA for factorial design followed by a Bonferroni post-test. ** *p* < 0.01 and **** *p* < 0.0001; *n* = 6–8 per group.

Chronic Ang II administration impairs NVC through NOX2-dependent oxidative stress [5]. Thus, since IL-17A neutralization and IL-17A receptor inhibition prevent NVC impairment and oxidative stress induced by chronic systemic administration of Ang II, we tested whether superoxide anions mediate the NVC impairment induced by IL-17A. We first evaluated the efficiency of Tempol, an antioxidant superoxide scavenger and superoxide dismutase-mimetic, and NOX2 deletion to normalize the superoxide anion production induced by IL-17A. Figure 5 showed a significantly higher production of superoxide anion in the somatosensory cortex (*p* < 0.0001) and the hippocampus (*p* < 0.01) in mice receiving the IL-17A Rb. In those regions, Tempol prevented this increase without modulating the superoxide levels in the control group. 

**Figure 6:**
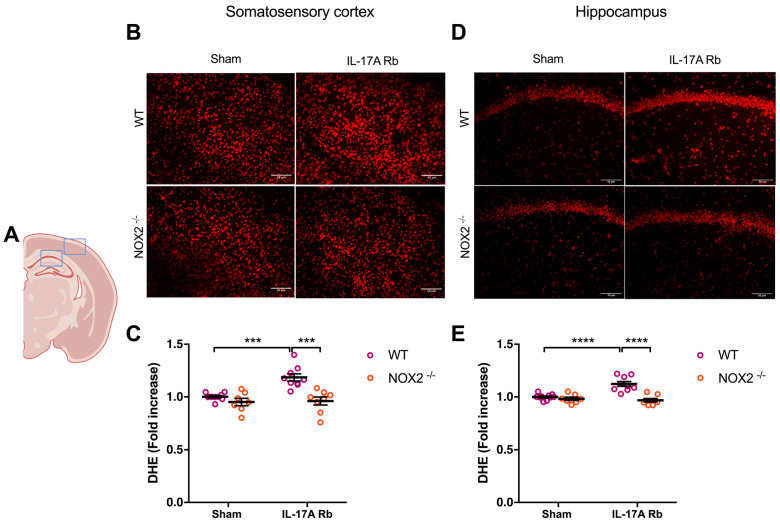
**NOX2 deletion prevents oxidative stress production induced by IL-17A**. Superoxide anion production was quantified using DHE in C57BL/6WT or NOX2^−/−^ male mice receiving IL-17A Rb through an osmotic minipump (50 pg/kg/h, 7 days). (**A**) the acquisition area representing the somatosensory cortex (right box) and the CA1 of the hippocampus (left box). Representative micrographs of DHE staining in (**B**) the somatosensory cortex and (**D**) the CA1 of the hippocampus (50 μm scale). The graphs depict the ratio of fluorescence compared to control in the (**C**) somatosensory cortex and (**E**) the hippocampus. Data were analyzed using an ANOVA for factorial design followed by a Bonferroni post-test. *** *p* < 0.001 and **** *p* < 0.0001; *n* = 7–10 per group.

Similarly, NOX2^−/−^ mice that received IL-17A Rb presented a similar level of superoxide anion production in the somatosensory cortex (*p* < 0.001) and the hippocampus (*p* < 0.0001) compared to the control mice (Figure 6). 

**Figure 7:**
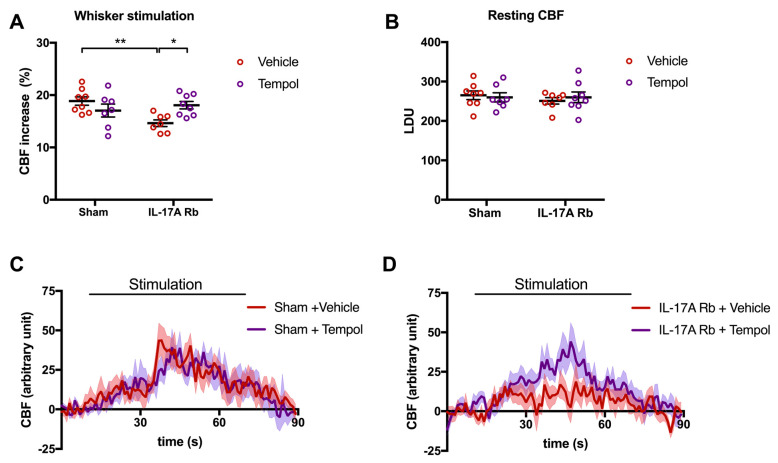
**Tempol treatment prevents the neurovascular coupling alteration induced by IL-17A**. CBF in response to whiskers stimulations measured in vivo by laser Doppler flowmetry in C57BL/6 male mice receiving IL-17A Rb through an osmotic minipump (50 pg/kg/h, 7 days) and treated with or without Tempol (1 mM changed every 2 days) administered in the drinking water. The graphs depict (**A**) CBF as percentage changes with respect to the initial CBF value, (**B**) resting CBF value as LDU, and (**C**,**D**) 1 s average curves of the evoked CBF, expressed in arbitrary unit. SEM is represented by the lighter tone shade surrounding each curve. Data were analyzed using an ANOVA for factorial design with repeated measures followed by a Bonferroni post-test. * *p* < 0.05 and ** *p* < 0.01; *n* = 7–8 per group.

These results are complementary to those observed in Figure 7, where the disruption of NVC by the IL-17A Rb was prevented by the Tempol treatment (*p* < 0.05). Tempol alone did not modulate CBF responses (Figure 7A,C). The relative resting CBF was similar in all groups (Figure 7B).

**Figure 8:**
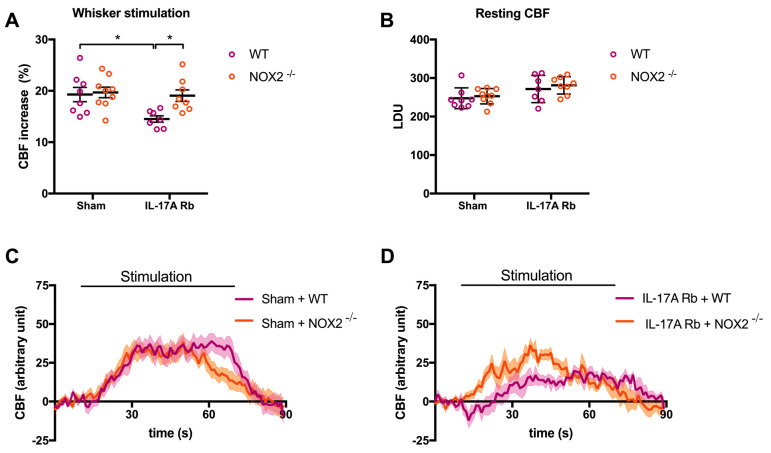
**NOX2 deletion prevents IL-17A-induced neurovascular coupling impairment.** CBF in response to whiskers stimulations measured in vivo by laser Doppler flowmetry in C57BL/6 WT or NOX2^−/−^ male mice receiving IL-17A Rb through an osmotic minipump (50 pg/kg/h, 7 days). The graphs depict (**A**) CBF as percentage changes with respect to the initial CBF value, (**B**) resting CBF value as LDU, and (**C**,**D**) 1 s average curves of the evoked CBF, expressed in arbitrary unit. SEM is represented by the lighter tone shade surrounding each curve. Data were analyzed using an ANOVA for factorial design followed by a Bonferroni post-test. * *p* < 0.05; *n* = 7–9 per group.

In the same manner, NOX2 deletion prevented NVC impairment induced by IL-17A Rb administration (Figure 8A,C,D; *p* < 0.05) without any difference in the laser Doppler perfusion units between the four groups (Figure 8B). Results from Supplemental Figure S4 showed that the deletion of the NOX2 gene did not prevent the increase in SBP observed at day 7 in response to IL-17A Rb administration (151.4 mmHg in C57BL/6 WT vs. 152.0 mmHg in NOX2^−/−^ mice). 

## 3. Discussion

We tested the hypothesis of whether IL-17A mediates the NVC impairment induced by Ang II through NOX2-derived ROS. The major new findings of this study are that neutralizing IL-17A or its receptor prevents Ang II-induced ROS production and NVC impairment. This was supported by showing ROS increase and NVC impairment following chronic IL-17A administration. A ROS scavenger and NOX2 deletion prevented these effects suggesting that NOX2-derived ROS production is responsible for the NVC impairment induced by this cytokine. 

Ang II through the Ang II type 1 receptor (AT1R) signaling pathway is an important pro-inflammatory stimulus, triggering the production of cerebral and systemic pro-inflammatory cytokines [4,17,18,19], chemokines [20] and ROS [5,11,21,22,23,24]. A putative role of inflammation in the effect of Ang II on NVC emerged in a study showing that the adaptive transfer of Treg lymphocytes (CD4+/CD25+) or of IL-10 prevents NVC impairment. In that study, Ang II administration increased the production of circulating pro-inflammatory cytokines such as IL-1α, IL-6, IL-17A, TNF-α, and LIF [4].

Inversely, a systemic inflammatory state in mice characterized by higher circulating IL-17A levels and induced by a high-salt diet contributed to NVC impairment [15]. In the present study, neutralizing IL-17A or inhibiting its receptor prevented the NVC impairment observed in an Ang II slow pressor hypertension model. Overall, these results suggest an important contribution of IL-17A in NVC impairment in models of hypertension. 

A lower increase in blood pressure induced by Ang II was observed in mice receiving IL-17A Ab or IL-17RA mAB [14] or in IL-17^−/−^ mice [11]. Our results confirmed the lower rise in blood pressure in mice receiving these Ab treatments. However, these slight changes in blood pressure most probably do not explain the NVC impairment induced by Ang II since it was previously demonstrated that the impact of Ang II on NVC is independent of its hypertensive effect [5,6,8].

In the periphery, IL-17^−/−^ mice presented preserved vascular functions, decreased superoxide production, and reduced T-cell infiltration in response to Ang II [11]. Therefore, we hypothesized that IL-17A could mediate the Ang II-induced NVC impairment by increasing oxidative stress. Our results confirm an increased superoxide anion production in the brain (somatosensory cortex and hippocampus) induced by Ang II. Interestingly, neutralizing IL-17A or inhibiting its receptor normalizes the superoxide anion production. These results are coherent with the mediating effects of IL-17A on peripheral blood vessels at the same regimen of Ang II administration as observed by Madhur et al. [11]. Overall, these findings suggest that IL-17A is involved in the superoxide anion production induced by Ang II, which may be a missing puzzle piece in the mechanism by which Ang II impairs NVC. 

To demonstrate that IL-17A can, by itself, impair the cerebrovascular response, we tested whether IL-17A Rb impairs NVC. We first showed that IL-17A Rb administration impaired NVC in a dose-dependent manner, where the 50 pg/kg/h dose showed a decrease in CBF in response to whiskers stimulations to a similar extent to the one seen in Ang II-induced hypertensive mice. The chosen concentration of IL-17A Rb seems to correspond to physiopathological levels observed in humans. In hypertensive patients, the concentrations vary between 1.3 pg/mL [25] and 14.5 pg/mL in refractory hypertensive patients [26]. In the present study, plasma IL-17A levels reached 13.4 and 11.1 in the Ang II and IL-17A groups, respectively. It is, nonetheless, worth mentioning that IL-17A levels in the plasma of hypertensive participants substantially vary with the duration of hypertension, the antihypertensive medication, and comorbidities [25]. Furthermore, no study has established the link between IL-17A levels and end-organ damages. Thus, further studies with large clinical cohorts will be necessary to establish the levels of IL-17A associated with cerebrovascular dysfunctions. 

In the present study, mice receiving Ang II or IL-17A Rb did not show an increased level of brain IL-17A compared to the control group. An absence of IL-17A increase in the brain was also observed in a high-salt diet model of hypertension despite an increase in the circulating IL-17A. Thus, circulating IL-17A may interfere with NVC by acting on cerebral endothelial cells or by communicating with the neurovascular unit from the meninges [15,27,28]; both mechanisms could ultimately lead to higher NOX2-derived superoxide production and cerebrovascular dysfunction [15,28]. In addition to ROS production, IL-17A may cause endothelial dysfunction via Rho-kinase activation [29].

Ang II impairs NVC through an increase in NOX2-derived superoxide anion [30]. Therefore, we investigated the role of oxidative stress in IL-17A-induced NVC dysfunction. In this study, mice were treated with the antioxidant Tempol due to its ability to cross membranes easily and its stronger therapeutic effect compared with other frequently used antioxidants [31]. Tempol prevented the increase in cerebral superoxide anion production in mice receiving IL-17A Rb. This effect was accompanied by a normalized CBF response to neuronal activation. These results suggest that increased superoxide anion production is a key mediator by which IL-17A impairs NVC. Superoxide anion can react with nitric oxide to form the highly reactive oxidant peroxynitrite. This mechanism is also implicated in the alteration of NVC by Ang II [10]. However, the role of peroxynitrite in the IL-17A-induced NVC alteration remains to be established. 

NADPH oxidase, specifically NOX2, the isoform expressed in cerebral endothelial cells, perivascular macrophages, microglia, and astrocytes, is likely the main source of increased cerebral superoxide anion in Ang II-induced hypertension in mice [5,10,24].Moreover, NOX2^−/−^ mice are protected from the Ang II-induced NVC alteration [5], further confirming the role of oxidative stress on cerebrovascular dysfunctions. We thus investigated the importance of NOX2 on cerebrovascular dysfunction and oxidative stress in response to IL-17A. We showed that IL-17A increases superoxide anion production. This increase was prevented by treatment with Tempol or in NOX2^−/−^ mice. Interestingly, NOX2 expression in the cortex and the hippocampus remained the same in mice receiving chronic Ang II or the IL-17A Rb administration compared to the control group (Supplemental Figure S5). These results suggest that IL-17A increases superoxide anion production by modulating NOX2 activity. NVC impairment caused by IL-17A is also mediated by NOX2 since similar CBF responses to whiskers stimulations were observed in NOX2^−/−^ mice compared with their corresponding wildtypes. This coincides with results observed in mouse aortic vascular smooth cells where IL-17A induces superoxide anion formation through NOX2 activation [16]. 

Finally, even though peripheral cardiovascular protection is possible, there was no difference in blood pressure between NOX2^−/−^ mice and their wildtypes. This supports previous results where NOX2^−/−^ mice receiving similar doses of Ang II as in the present study (764 ng/kg/min) do not present a lower SBP [32]. 

**Figure 9:**
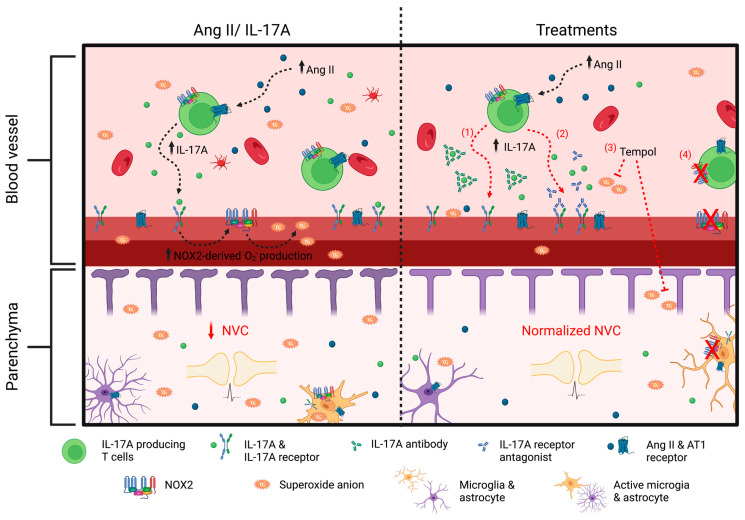
**Interleukin-17A, through NOX2-derived superoxide anion production, is a modulator of neurovascular coupling impairment induced by angiotensin II.** Increased angiotensin (Ang II) in hypertension leads to an inflammatory state with higher concentrations of circulating interleukin (IL)-17A. The latter, through NOX2-derived superoxide anion production, is an important mediator of neurovascular coupling (NVC) impairment induced by Ang II. (**1**) Neutralizing IL-17A or (**2**) inhibiting its receptor prevents Ang II-induced increased reactive oxygen species (ROS) production and neurovascular decoupling. Similarly, a treatment with (**3**) Tempol and (**4**) NADPH oxidase (NOX) 2 gene deletion prevents ROS production and NVC impairment induced by IL-17A. These findings suggest that IL-17A, through superoxide anion production, is an important mediator of cerebrovascular dysregulation induced by Ang II and could be a potential therapeutic target to prevent cerebrovascular dysfunction in hypertensive patients.

In conclusion, we have demonstrated that IL-17A, through superoxide anion production, is an important modulator of NVC impairment induced by Ang II. Altogether, our findings suggest that modulating the immune system and targeting inflammation in hypertension could be a promising approach for reducing cerebrovascular dysfunctions (Figure 9). Given that hypertension and chronic inflammation are important risk factors for stroke, vascular cognitive impairment, and Alzheimer’s disease, the results of this study could open the door for future investigations to examine the influence of the immune system and inflammation on brain degeneration.

## 4. Materials and Methods

### 4.1. Animals

The study was approved by the Committee on Ethics of Animal Experiments of the Université de Montréal and performed in accordance with the guidelines of the Canadian Council for Animal Care and by the ARRIVE (Animal Research: Reporting of In Vivo Experiments). Ten- to twelve-weeks-old C57BL/6 male mice from Charles River Laboratories (Saint-Constant, Qc, Canada) were individually housed in a temperature-controlled room with ad libitum access to water and a standard protein rodent diet (Tekland global 18% protein rodent diet). Ten-weeks-old C57BL/6 male mice with a targeted genetic deletion of NOX2 (B6.129S-Cybbtm1Din/J; stock No: 002365) and their controls were obtained from Jackson Laboratory (Bar Harbord, ME, USA). Given that the female mice are protected from the deleterious effects of Ang II on cerebrovascular functions [23], only male mice were used in this study. Following acclimatization, animals were randomly assigned to experimental groups.

### 4.2. Drugs Administration

Osmotic minipumps (model 1002; Alzet, Cupertino, CA, USA) containing Ang II (MilliporeSigma, Oakville, ON, Canada) were subcutaneously implanted under isoflurane anesthesia as previously described [33]. Briefly, mice received bupivacaine hydrochloride (Marcaine; CDMV, Canada, 2 mg/kg s.c.) at the site of the incision before the osmotic pump implantation. Each osmotic pump delivered 600 ng/kg/min of Ang II for 14 days while the control group was sham-operated. NVC impairment induced by Ang II between sham-operated mice and mice receiving saline through an osmotic pump was compared in a separate group of experiments. The mice were injected i.p. every four days with an IL-17A neutralizing antibody (0.5 µg/µL; eBioMM17F3; eBioscience – Thermo Fisher Scientific, Burlington, ON, Canada), a specific IL-17A receptor antagonist (0.5 µg/µL; PL-31280; Amgen, Thousand Oaks, CA, USA), or an immunoglobulin G (IgG) isotype control (0.5 µg/µL Invitrogen – Thermo Fisher Scientific, Burlington, ON, Canada) starting on the day of the implantation (Supplemental Figure S6A). This administration regimen was chosen based on prior studies on murine models of hypertension and atherosclerosis [14,34,35].

In another group of animals, systemic infusion of 50 pg/kg/h of mouse-recombinant IL-17A (IL-17A Rb; 421-ML/CF; R&D system, Minneapolis, MN, USA) for 7 days was achieved via an osmotic minipump (model 1007D; Alzet) (Supplemental Figure S6B). Since no study has previously shown the effect of systemic infusion of IL-17A on NVC, a dose-response curve of IL-17A Rb on cerebrovascular responses was assessed. IL-17A Rb administration has shown a dose-dependent effect on CBF in response to whiskers stimulations. The 50 pg/kg/h dose was chosen because it showed a decrease in cerebrovascular response to the level seen in Ang II-induced hypertensive mice (Supplemental Figure S3). A subgroup of C57BL/6 mice was simultaneously treated with Tempol (4-hydroxy-TEMPO; Millipore Sigma,Oakville, ON, Canada; 1 mmol/L) dissolved in drinking water or with its vehicle (regular drinking water). Treatment with Tempol started 2 days before the osmotic pump implantation and ended at the time of sacrifice (one week after surgery) (Supplemental Figure S6C).

### 4.3. Systolic Blood Pressure Monitoring

SBP was monitored in awake mice using tail-cuff plethysmography (Kent Scientific Corp, Torrington, CT, USA). Mice were warmed on a heating pad preheated at 37 °C for ten minutes before and during blood pressure recordings. Animals were habituated to the procedure three days before blood pressure assessment. Right before the implantation of osmotic minipumps (day 0) and weekly until the NVC analysis, ten blood pressure assessments per mouse were measured and averaged. Blood pressure was monitored by the same person at the same period of the day.

### 4.4. Neurovascular Coupling

Anesthesia was initiated with isoflurane (induction: 5%, maintenance: 2%) and maintained with 50 mg/kg of α-chloralose i.p. (MilliporeSigma, Oakville, ON, Canada) and 750 mg/kg of urethane i.p. (MilliporeSigma, Oakville, ON, Canada). The depth of anesthesia was checked by testing corneal reflexes and motor responses to tail pinch. Mean blood pressure and blood sample collection for gas assessment were monitored through the catheterization of the femoral artery. Mice were artificially ventilated with a nitrogen/oxygen/CO_2_ mixture through tracheal intubation. Body temperature was maintained at 37 °C throughout the experiment. CBF was monitored with a laser Doppler probe (AD Instruments, Colorado Springs, CO, USA) placed on the thinned skull above the whisker-barrel area of the somatosensory cortex. The flowmeter and blood pressure transducer were connected to a computerized data acquisition system (MacLab; Colorado Springs, CO, USA). Analysis of CBF responses began 30 min after the end of the surgery to allow blood gases to stabilize. Animals with mean arterial blood pressure under 60 mmHg and/or blood gases outside the normal range (pH: 7.35–7.40; pCO_2_: 33–45; and pO_2_: 120–140) were eliminated from the study. CBF responses to neuronal activity were evaluated during whiskers stimulations. Three whiskers stimulations sessions of one minute were performed on the contralateral side of the CBF measurement. Three minutes of resting periods were left between each stimulation. CBF values were acquired with the LabChart6 Pro software (v6.1.3, AD Instruments, Colorado Springs, CO, USA). The percentage increase in CBF represents the peak CBF response relative to the resting CBF peak values during the 20 s before stimulations.

### 4.5. Superoxide Anion Production

Superoxide anion production was assessed by hydroethidine microfluorography as previously described [36]. Hydroethidine (dihydroethidium) is cell permeable and is oxidized to become the fluorophore ethidium bromide that intercalates in double-stranded DNA [37]. Mice were anesthetized with sodium pentobarbital (100 mg/kg body weight, CDMV, Saint-Hyacinthe, Qc, Canada) and transcardially perfused with Phosphate-buffered saline (PBS) 1X, pH 7.4. Brains were carefully isolated, frozen on dry ice, and stored at −80 °C until further analysis. Frozen brains were cut with a cryostat (20 µm), and brain sections were mounted on slides and stored at −20 °C overnight. The slides were air dried at room temperature for 15 min followed by 15 min on a slide warmer set at 45 °C. The slides were then immersed in a dihydroethidium (DHE) solution (2 µM, MilliporeSigma, Oakville, ON, Canada) dissolved in PBS 1X at 37 °C for 2 min. The slides were rinsed in PBS for 5 min and dried on a slide warmer for 20 min before they were coverslipped with a Fluoromount-G mounting medium (Southern Biotech, Birmingham, AL, USA). Images were acquired in the somatosensory cortex and the hippocampus (average analysis of the lacunosum moleculare (LMol), the dentate gyrus (DG), the cornu ammonis 1 (CA1), and the cornu ammonis 3 (CA3)) using an epifluorescence microscope Leica DM2000 with the same acquisition parameters for all groups. Analysis of relative fluorescence intensity was conducted using the ImageJ software (version 1.53; National Institutes of Health). Briefly, following subtracting the background for all micrographs, the mean fluorescence intensity was measured. A ratio of the mean intensity for each mouse relative to the mean intensity of the daily controls was calculated to avoid possible variability between days of the experiment. As such, DHE results are expressed as a ratio relative to the control group for each set of experiments.

### 4.6. NOX2 Expression

Hippocampal and cortex tissue lysates were prepared using a lysis buffer (Tris 50 mM, NP-40 1%, NaCl 137 mM, glycerol 10%, MgCl_2_ 5 mmol/L, sodium fluoride 20 mM, sodium pyrophosphate 1 Mm, sodium orthovanadate 1 mM, pH 7.4) complemented with a protease inhibitor EDTA-free tablet (MilliporeSigma, Oakville, ON, Canada). Proteins (25 µg) were loaded and run on polyacrylamide gels (10%) and then transferred onto nitrocellulose membranes (Biorad, Saint-Laurent, Qc, Canada). The transferred proteins were detected using the specific primary antibodies anti-NOX2 (ab129068, Abcam, Toronto, ON, Canada) and Pan-Actin as a loading control (4968S, Cell Signaling Technology, Danvers, MA, USA) at a concentration of 1:5000 in Tris-Buffered Saline-Tween (TBST; Tris 20 mM, NaCl 137 mM, Tween-20 0.1%, pH 7.6) containing 5% skim milk and 1:1000 in TBST containing 5% BSA, respectively. The secondary antibody was an HRP-linked antibody (7074, Cell Signaling Technology, Danvers, MA, USA) used at a concentration of 1:5000 in TBST containing 5% skim milk and 1:10,000 in TBST containing 5% BSA, respectively. Chemiluminescence was used to detect protein expression, and membranes were digitalized using a GE LAS 4000 mini. Band intensities (integrated optical density) were quantified with the ImageJ software (version 1.53; National Institutes of Health). NOX2 results are expressed relative to the Pan-Actin loading control. 

### 4.7. Brain Homogenate and Plasmatic IL-17A Levels

Brains were homogenized in PBS 1X complemented with a protease inhibitor EDTA-free tablet (MilliporeSigma, Oakville, ON, Canada). Brain homogenates and plasma samples were sent to Eve Technologies Corporation, where a Mouse High Sensitivity T-Helper Cells Custom Assay (Eve Technologies Corporation, Calgary, AB, Canada) was used for the quantitative analysis of mouse IL-17A. 

### 4.8. Statistical Analysis

Data analysis was performed with GraphPad Prism software (version 7.0, La Jolla, USA), and results are presented as mean ± SEM. CBF responses to whiskers stimulations, resting CBF, superoxide anion production, and SBP analysis were evaluated with an ANOVA for factorial design with repeated measures followed by a Bonferroni post-test for multiple group comparisons. The dose-response effect on CBF increases in response to whiskers stimulations, relative resting CBF, and mean arterial pressure, as well as the NOX2 expression and IL-17A plasmatic levels, were analyzed using a one-way ANOVA followed by a Dunnet’s post-test comparing each group with the Sham group. Significance was set at *p* < 0.05. Sample size per group is presented in the results section as well as in the figure legends.

## Data Availability

The data used to support the findings of this manuscript are available on request to the corresponding author.

## Supplementary Materials

The following supporting information can be downloaded at: https://www.mdpi.com/article/10.3390/ijms24043959/s1.
