# Supplementary material for: Neurovascular Coupling in Hypertension Is Impaired by IL-17A through Oxidative Stress
Source: Int J Mol Sci. 2023 Feb 16;24(4):3959. doi: 10.3390/ijms24043959 (PMC9967204; doi:10.3390/ijms24043959)
Supplement: Supplementary file 1 [file ijms-24-03959-s001.zip › ijms-2147313-supplementary.pdf]

Supplemental Materials

Neurovascular Coupling in hypertension is impaired by IL-17A through Oxidative Stress

J. YOUWAKIM[1,2,3], D. VALLERAND[1,2] and *H. GIROUARD[1,2,3,4]

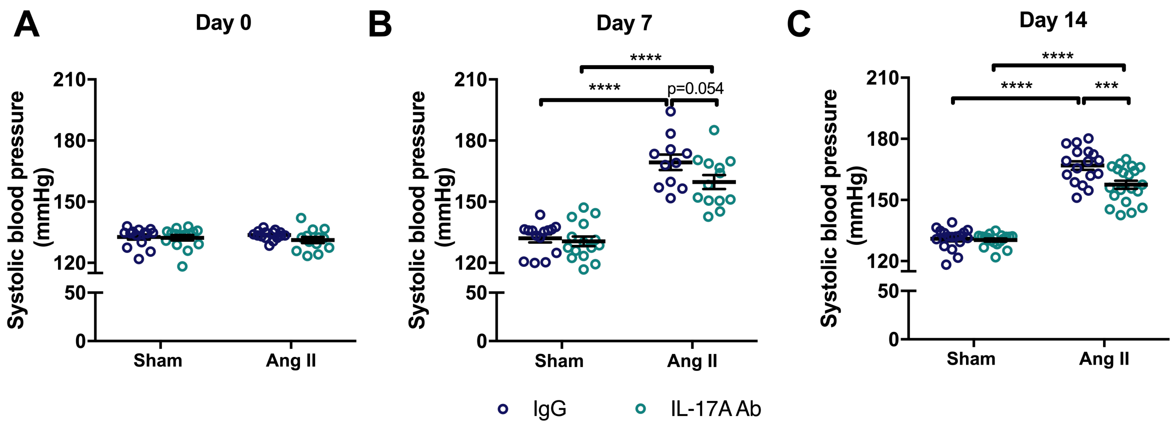

Supplemental Figure S1: IL-17A neutralization partially prevents the increased systolic blood pressure induced by Ang II. Systolic blood pressure assessed at (A) day 0, (B) day 7 and (C) day 14 by tail-cuff plethysmography in C57BL/6 male mice treated with an IL-17A Ab or control mouse IgG antibody (0.5 µg/µL per mouse every 4 days, i.p.). This administration started at day 0 of Ang II (600 ng/kg/min, 14 days) infusion through an osmotic minipump or sham surgery. Data were analysed using ANOVA for factorial design followed by a Bonferroni post-test., ***p<0.001 and ****p<0.0001; n=11-18 per group.

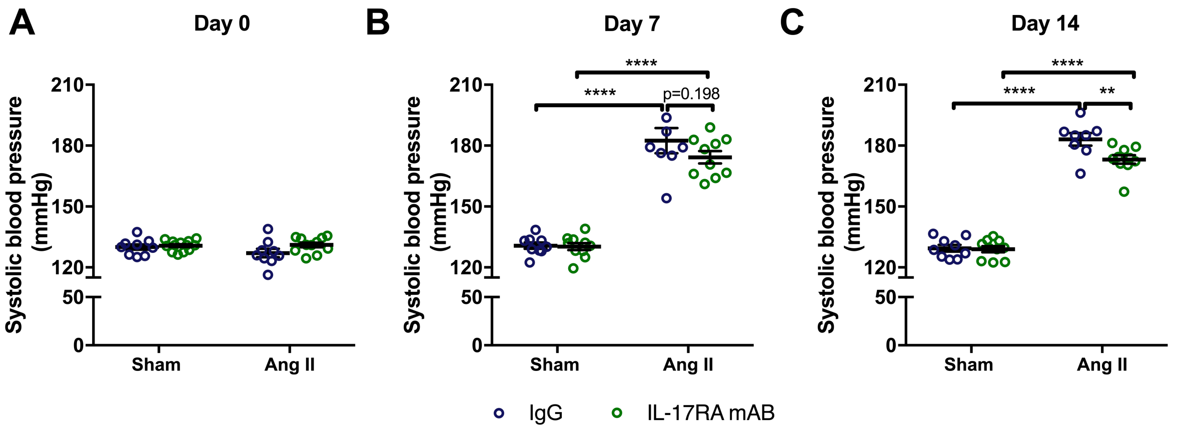

Supplemental Figure S2: IL-17A receptor inhibition partially prevents increased systolic blood pressure induced by Ang II. Systolic blood pressure assessed at (A) day 0, (B) day 7 and (C) day 14 by tail-cuff plethysmography in C57BL/6 male receiving an IL-17A receptor antagonist (IL-17RA mAB) or control mouse immunoglobulin G (IgG) antibody (0.5 µg/µL per mouse every 4 days, i.p.). This administration started at day 0 of Ang II (600 ng/kg/min, 14 days) infusion through an osmotic minipump or sham surgery. Data were analysed using ANOVA for factorial design followed by a Bonferroni post-test. **$p<0.01$ and ****$p<0.0001$; n= 9-13 per group.

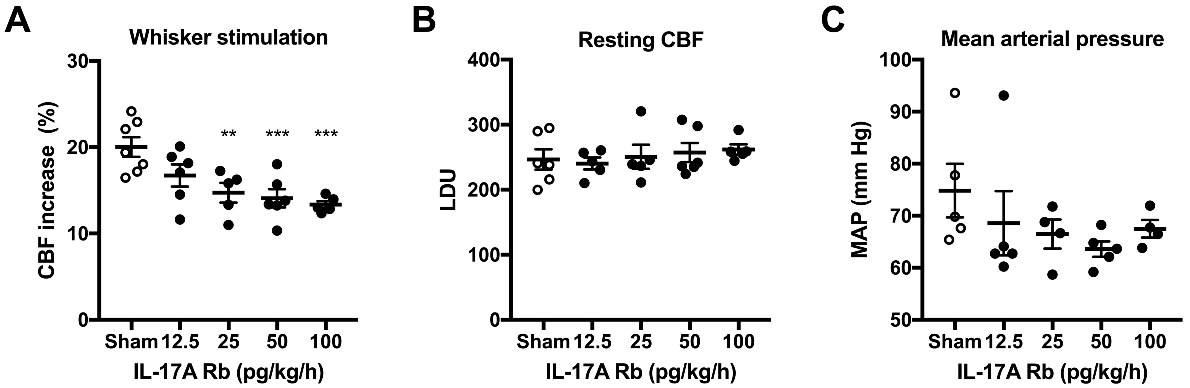

Supplemental Figure S3: Effect of chronic systemic recombinant IL-17A administration on neurovascular coupling. CBF responses to whiskers stimulations measured *in vivo* by laser Doppler flowmetry in C57BL/6 male mice receiving 12.5 to 100 pg/kg/h of IL-17A Rb through an osmotic minipump (7 days) or sham surgery. The graphs depict (A) CBF as percentage changes with respect to the initial CBF value, (B) the resting CBF value as LDU, or (C) the mean arterial pressure monitored simultaneously with CBF. Data were analysed using one-way-ANOVA followed by Dunnet's post-test comparing each group with the Sham group. **p<0.01, *** p<0.001; n=5-7 per group.

Supplemental Table S1: Quantitative analysis of IL-17A in brain homogenate and plasma.

	Treatment	*IL-17A (pg/mL)*	*p-value*
Brain Homogenate	Sham	0.80 ± 0.03	-
	Ang II	0.81 ± 0.06	0.98
	IL-17A Rb	0.85 ± 0.08	0.75
Plasma	Sham	7.66 ± 0.80	-
	Ang II	13.43 ± 3.75	0.26
	IL-17A Rb	11.12 ± 2.60	0.58

Data depicted as mean ± SEM were analysed using one-way-ANOVA followed by Dunnet's post-test comparing each group with the Sham group; n = 6 per group for brain homogenate; n=11-13 per group for plasma sample.

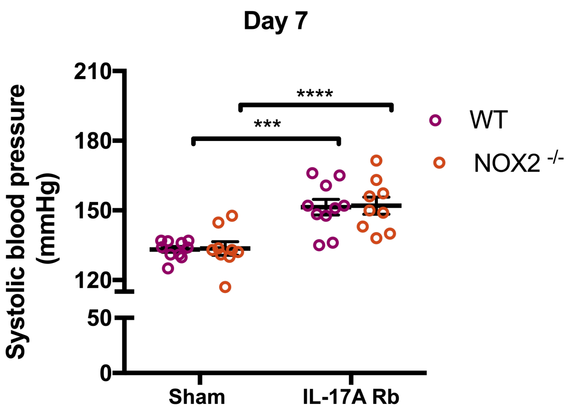

Supplemental Figure S4: NOX2 deletion doesn't prevent systolic blood pressure increase induced by chronic IL-17A Rb administration. Systolic blood pressure assessed at day 7 by tail-cuff plethysmography in C57BL/6 WT or NOX2 $^{-/-}$ male mice receiving IL-17A Rb through an osmotic minipump (50 pg/kg/h, 7 days). Data were analysed using ANOVA for factorial design followed by a Bonferroni post-test. ***p<0.001, ****p<0.0001; n=8-10 per group.

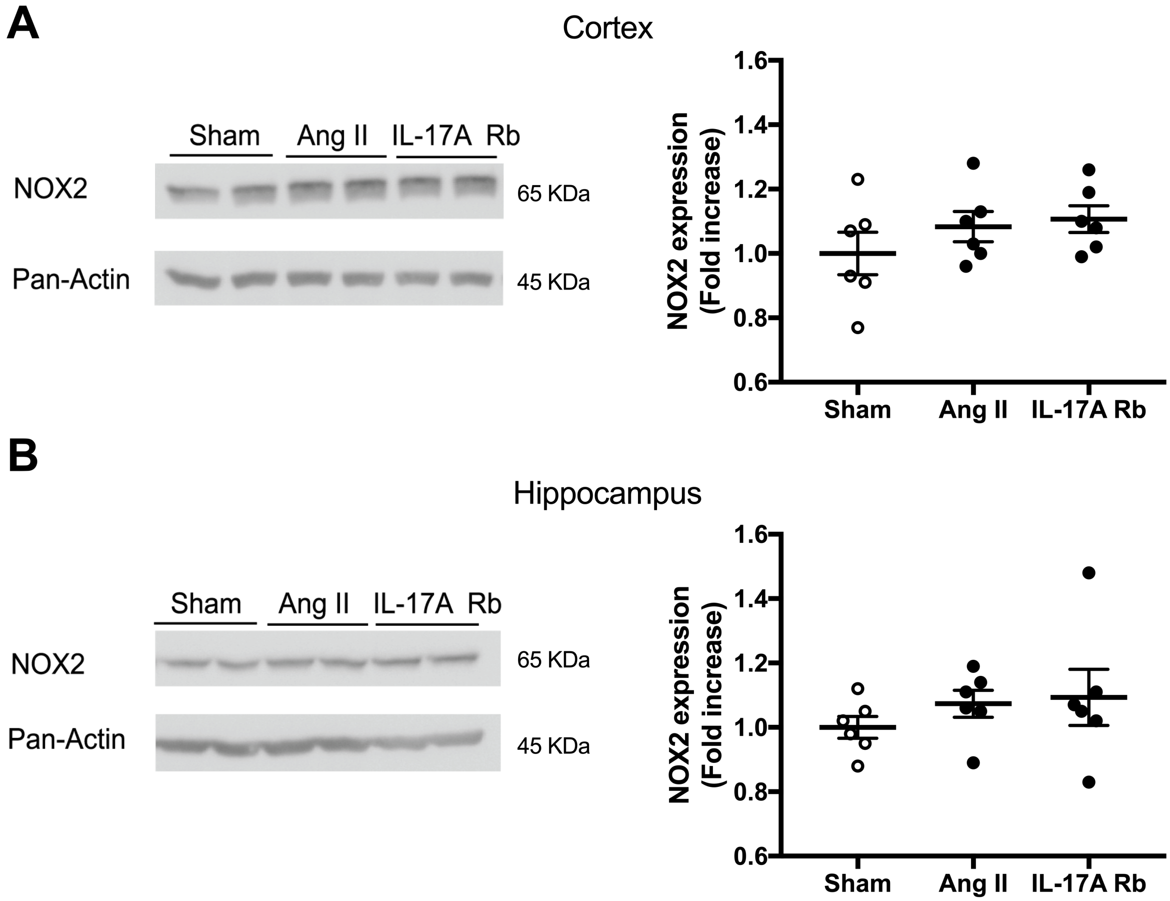

Supplemental Figure S5: NOX2 expression does not significantly change following chronic Ang II and IL-17A Rb administration. Western blot analysis of NOX2 expression in the (A) cortex and (B) hippocampus in C57BL/6 mice receiving IL-17A Rb (50 pg/kg/h, 7 days) or Ang II (600 ng/kg/min, 14 days through an osmotic minipump or Sham surgery. Data were analysed using one-way-ANOVA followed by Dunnet's post-test comparing each group with the Sham group; n= 6 per group.

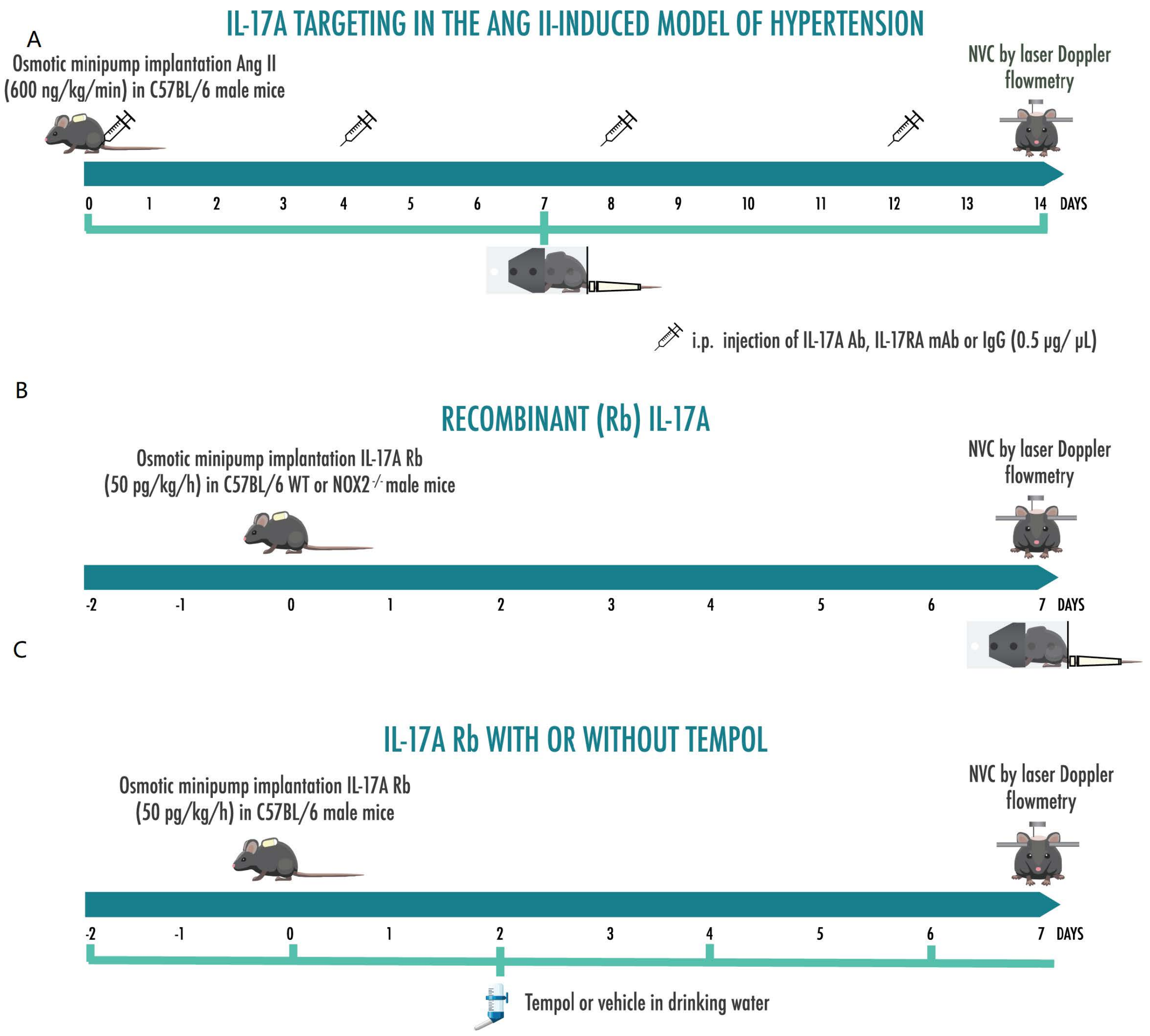

Supplemental Figure S 6: Schematic representation of experimental animal protocol. (A) C57BL/6 male mice treated with IL-17A Ab, IL-17RA mAB or the control mouse IgG antibody and receiving or not Ang II. SBP was measured on day 0, 7 and 14 and NVC was assessed on day 14. (B) 7 day IL-17A Rb administration in C57BL/6 WT and NOX2$^{-/-}$ mice. SBP and NVC were assessed on day 7. (C) C57BL/6 mice receiving or not IL-17A Rb and treated with or without Tempol administered in the drinking water. NVC were assessed on day 7.
